# Supplementary material for: Empirical methods for controlling false positives and estimating confidence in ChIP-Seq peaks
Source: BMC Bioinformatics. 2008 Dec 5;9:523. doi: 10.1186/1471-2105-9-523 (PMC2628906; doi:10.1186/1471-2105-9-523)
Supplement: Additional File 1 — A variety of html documents from the USeq web site detailing the available applications, their best usage, output file type descriptions, command line menus, etc. [file 1471-2105-9-523-S1.zip › USeqUserGuides/JavaDoc.zip!/JavaDoc/trans/roc/ParsePatternedWindows.html]

ParsePatternedWindows


|  |  |  |
| --- | --- | --- |
| **Overview** \| **Package** \| **Class** \| **Use** \| **Tree** \| **Deprecated** \| **Index** \| **Help** | |  |
| **PREV CLASS**   **NEXT CLASS** | **FRAMES**    **NO FRAMES**     **All Classes** |  |
| SUMMARY: NESTED \| FIELD \| CONSTR \| METHOD | DETAIL: FIELD \| CONSTR \| METHOD |  |


---


## trans.roc Class ParsePatternedWindows

```
java.lang.Object
  trans.roc.ParsePatternedWindows
```

---

``` public class ParsePatternedWindows extends java.lang.Object ```

Finds RocWindows upstream and withing the neighborhood of the ATG start site of a patterned gene list.

---

**Constructor Summary**

`ParsePatternedWindows(java.lang.String[] args)`


| **Method Summary** | |
| --- | --- |
| `static GeneGroup[]` | `extractGeneGroups(java.util.HashMap geneGroups, java.lang.String[] geneList)`             Pulls out a sub set of GeneGroups with the names in the geneList. |
| `static RocWindow[]` | `fetchProximalWindows(GeneGroup[] geneGrps, RocWindow[] windows, int sizeUpstream)` |
| `static RocWindow[]` | `filterRocWindows(RocWindow[] windows, double cutOff, boolean greaterThan)`             Scans an array of RocWindow for those that are greater than (or less than if boolean is false) or equal to the cutOff. |
| `static RocWindow[]` | `loadRocWindows(java.io.File[] arrayLists)`             Loads all the RocWindows into one from serialized ArrayLists of RocWindows. |
| `static RocWindow[]` | `loadTxtRocFile(java.io.File file)`             reads a txt roc window (chrom, start, stop, middle(not used), score). |
| `static void` | `main(java.lang.String[] args)` |
| `static void` | `printChroms(RocWindow[] r)` |
| `static void` | `printDocs()` |
| `void` | `processArgs(java.lang.String[] args)`             This method will process each argument and assign new varibles |
| `static void` | `writeSGRFile(RocWindow[] r, java.io.File file)`             Writes an sgr file. |
| `static void` | `writeTxtRocFile(RocWindow[] r, java.io.File file)`             Writes a chrom, start, stop, score file. |

**Methods inherited from class java.lang.Object**

`equals, getClass, hashCode, notify, notifyAll, toString, wait, wait, wait`

**Constructor Detail**

### ParsePatternedWindows

```
public ParsePatternedWindows(java.lang.String[] args)
```


**Method Detail**

### writeSGRFile

```
public static void writeSGRFile(RocWindow[] r,
                                java.io.File file)
```

:   Writes an sgr file.

---


### writeTxtRocFile

```
public static void writeTxtRocFile(RocWindow[] r,
                                   java.io.File file)
```

:   Writes a chrom, start, stop, score file.

---


### loadTxtRocFile

```
public static RocWindow[] loadTxtRocFile(java.io.File file)
```

:   reads a txt roc window (chrom, start, stop, middle(not used), score).

---


### printChroms

```
public static void printChroms(RocWindow[] r)
```

---


### filterRocWindows

```
public static RocWindow[] filterRocWindows(RocWindow[] windows,
                                           double cutOff,
                                           boolean greaterThan)
```

:   Scans an array of RocWindow for those that are greater than (or less than if boolean is false) or
    equal to the cutOff. Returns those that pass.

---


### loadRocWindows

```
public static RocWindow[] loadRocWindows(java.io.File[] arrayLists)
```

:   Loads all the RocWindows into one from serialized ArrayLists of RocWindows.

---


### extractGeneGroups

```
public static GeneGroup[] extractGeneGroups(java.util.HashMap geneGroups,
                                            java.lang.String[] geneList)
```

:   Pulls out a sub set of GeneGroups with the names in the geneList.

---


### fetchProximalWindows

```
public static RocWindow[] fetchProximalWindows(GeneGroup[] geneGrps,
                                               RocWindow[] windows,
                                               int sizeUpstream)
```

---


### printDocs

```
public static void printDocs()
```

---


### processArgs

```
public void processArgs(java.lang.String[] args)
```

:   This method will process each argument and assign new varibles

---


### main

```
public static void main(java.lang.String[] args)
```


---


|  |  |  |
| --- | --- | --- |
| **Overview** \| **Package** \| **Class** \| **Use** \| **Tree** \| **Deprecated** \| **Index** \| **Help** | |  |
| **PREV CLASS**   **NEXT CLASS** | **FRAMES**    **NO FRAMES**     **All Classes** |  |
| SUMMARY: NESTED \| FIELD \| CONSTR \| METHOD | DETAIL: FIELD \| CONSTR \| METHOD |  |


---
